# Quality of life and climacteric symptoms in women with endometrial cancer: examining the impact of lower limb lymphedema

**DOI:** 10.1186/s41687-025-00895-0

**Published:** 2025-06-13

**Authors:** Chia-Chun Li, Ting-Chang Chang, Chi-Wen Chang, Chun-Hsia Huang, Yun-Fang Tsai, Chiu-Lan Huang, Lynn Chen

**Affiliations:** 1 School of Nursing, College of Medicine, Chang Gung University, No. 259, Wenhua 1st Rd., Guishan Township, Taoyuan County, Tao-Yuan, 333 Taiwan; 2 College of Medicine, Chang Gung University, Tao-Yuan, Taiwan; 3 Department of Obstetrics and Gynecology, Linkou Chang Gung Memorial Hospital, Tao-Yuan, Taiwan; 4 Division of Pediatric Endocrinology & Genetics, Department of Pediatrics, Linkou Chang Gung Memorial Hospital, Tao-Yuan, Taiwan; 5 Department of Nursing, Linkou Chang Gung Memorial Hospital, Tao-Yuan, Taiwan; 6 Department of Nursing, Chang Gung University of Science and Technology, Tao-Yuan, Taiwan; 7 Department of Psychiatry, Chang Gung Memorial Hospital at Keelung, Keelung, Taiwan; 8 School of Nursing, University of Maryland at Baltimore, Baltimore, MD USA

**Keywords:** Quality of life, Climacteric symptoms, Lower limb lymphedema, Endometrial cancer

## Abstract

**Background:**

Women undergoing treatment for endometrial cancer (EC) often experience climacteric-like symptoms, and some may develop lower limb lymphedema (LLL). LLL can significantly impact women’s quality of life (QoL) and exacerbate climacteric symptoms. In this study, we investigated the prevalence of LLL and explored the differences in climacteric symptoms and QoL between women with and without LLL.

**Methods:**

A cross-sectional, observational, and comparative study design was employed. Clinical data for 105 women diagnosed with EC were gathered, encompassing demographic details, LLL, climacteric symptoms, and QoL. Instruments used included a demographic-disease survey, the Gynecological Cancer Lymphedema Questionnaire, the Greene Climacteric Scale, and EORTC QLQ-C30 and QLQ-EN24 questionnaires. Descriptive statistics and independent-sample *t*-tests were utilized for data analysis.

**Results:**

In this study, 39% of women with EC experienced LLL, with primary symptoms being aching, heaviness, and swelling. Women with LLL also had significantly more severe climacteric symptoms, including anxiety, depression, and vasomotor issues, and they reported poorer physical, role, emotional, cognitive, and social functioning. Additionally, they faced increased fatigue, pain, dyspnea, and more severe specific symptoms like lymphedema, urological and gastrointestinal issues, body image concerns, back/pelvic pain, and hair loss compared to those without LLL.

**Conclusion:**

The findings of this study enhance understanding of the impact of LLL on climacteric symptoms and QoL in women with EC. Health-care professionals, when advising treatment for EC, should inform women about the likelihood of LLL and assist in early management of its symptoms during and after EC treatment.

## Introduction

In 2020, endometrial cancer (EC) ranked as the sixth-most diagnosed cancer in women globally, with 417,000 new diagnoses [1]. In Taiwan, it was the fifth-leading cancer among women, with 3,032 diagnoses in the same year [2]. Since 1990, the incidence rates of EC have surged in many countries, particularly in high-income countries [3]. In Taiwan, the age-standardized incidence rate of EC has seen a nearly tenfold rise, from 1.72 per 100,000 in 1980 to 16.37 in 2020 [4]. Given the early onset of the most common symptom, vaginal bleeding, many Taiwanese women (66%) are diagnosed at stage 1 [2]. The five-year overall survival rate for Taiwanese women with EC is around 83% [5]. The two primary treatment modalities are surgery alone and surgery with adjuvant radiotherapy in early-stage EC [2]. The treatments may result in side effects, such as climacteric symptoms and lower limb lymphedema (LLL) [6, 7]. While these complications may not be life-threatening, they can significantly influence a woman’s quality of life (QoL) [8]. Given the rapid growth of this population, there’s a pressing need for researchers and health-care professionals to give these complications greater attention.

Women diagnosed with early-stage EC often undergo recommended surgical procedures such as total hysterectomy, bilateral salpingo-oophorectomy (BSO), and lymph node dissection [3]. Following a bilateral oophorectomy, premenopausal women undergo surgical menopause, which can result in symptoms such as vasomotor disturbances, mood disorders (such as depression and anxiety), sexual dysfunctions (such as reduced sexual desire), osteoporosis, cognitive impairments, sleep disturbances, and cardiovascular disease [9–11]. Vasomotor symptoms typically present as hot flashes and sweating [12]. Past research has shown that climacteric symptoms tend to intensify over time in women with EC [13]. The role of lymph node dissection in treating EC remains debated. One previous study, involving 42,184 women with EC, found that increased numbers of nodes dissected during lymph node dissection were associated with better overall survival [14]. However, a contrasting meta-analysis reported no significant differences in overall and recurrence-free survival between women who underwent lymphadenectomy and those who did not [15]. Furthermore, lymphadenectomy might increase the risk of postoperative morbidity and lymphedema [15].

In Taiwan, the increasing incidence of EC contrasts sharply with the scarcity of research on this population, particularly regarding the prevalence of LLL and the manifestation of climacteric symptoms following surgery and adjuvant therapies. A systematic review shows that the prevalence of LLL varies widely, from 0 to 50%, depending on the extent of lymph node removal and subsequent radiotherapy [16]. LLL impacts the appearance, sensation, and function of a woman’s lower limbs, presenting symptoms such as pain, fullness, heaviness, tightness, tingling, numbness, and aching [17], which significantly diminish QoL [7].

A previous quantitative study involving 639 women with EC assessed the impact of LLL or swelling (LLS) on QoL, revealing that women with LLL experienced significantly worse physical QoL as measured by the Medical Outcome Study Short Form-12 (SF-12), although their mental QoL remained within normative ranges [7]. Additionally, a mixed-method study of 132 women with EC found that those who underwent surgery alone achieved significantly better scores on the Functional Assessment of Cancer Therapy–General (FACT-G) compared to those who also received chemotherapy and radiation [18]. These women reported long-term treatment side effects such as perineal pain, easy fatigability, loss of appetite, and urinary or bowel issues, which severely impacted their daily activities. Concerns about cancer recurrence and financial insecurity also added to their emotional distress [18]. Moreover, a recent quantitative study with 221 women with EC used the European Organization for Research and Treatment of Cancer Quality of Life Questionnaire (EORTC QLQ-C30) and identified lower extremity lymphedema, especially when coupled with obesity, as a strong predictor of poorer QoL in women undergoing planned sentinel lymph node (SLN) staging for EC [19].

While extensive research exists on the effects of upper limb lymphedema on QoL in women with breast cancer [20], there is a significant research gap for women with EC, particularly regarding the impact of LLL. This deficiency is especially pronounced among Taiwanese women with EC, underscoring an urgent need for targeted research. The importance of QoL as a pivotal factor in clinical cancer research continues to grow, informing treatment decisions and emphasizing the need for comprehensive care approaches [18].

This observational study seeks to explore the prevalence of LLL and investigate climacteric-like symptoms among Taiwanese women with EC. We also aim to assess their QoL using the EORTC QLQ-C30 (version 3.0) [21] and Quality of Life Questionnaire–Endometrial Cancer Module (QLQ-EN24) [22]. Notably, there is limited research on how LLL influences climacteric symptoms in women with EC. Thus, this study also aims to explore the differences in climacteric symptoms and QoL between Taiwanese women with EC who are experiencing LLL and those who are not.

## Methods

### Design, sample, and setting

This cross-sectional, observational, comparative study is part of a broader research project examining the antecedents and mediators of physical activity in relation to QoL in women with EC. With the approval from the Institutional Review Board (IRB) at Chang Gung Medical Foundation (IRB Protocol 201600229B0), women diagnosed with EC from the outpatient gynecological oncology clinics at Linkou Chang Gung Memorial Hospital in North Taiwan between May 2017 and July 2020. Diagnoses were confirmed by their gynecological physicians. Convenience sampling was employed, and participants were selected based on the following inclusion criteria: (1) aged 20 years or older; (2) diagnosed with EC and aware of their diagnosis; (3) had completed cancer treatment at least six months prior to participation; (4) had no history of other cancer diagnoses; (5) were willing to provide informed consent and authorize the release of their health information; and (6) were able to read Mandarin Chinese. Women with current diagnoses of cognitive impairments or psychiatric disorders, other than depression, were excluded from the study.

### Procedure

Women with EC attending gynecological clinics for follow-up visits and meeting the eligibility criteria were referred to the research team by their gynecological oncologists. Interested women were approached by a skilled research assistant who discussed potential participation. After obtaining written informed consent, paper-based questionnaires were distributed to the women as they waited for their appointments in the clinic rooms. These questionnaires were administered only once; women who had previously participated were not eligible to complete them again. Additionally, women filled out demographic and related questionnaires. Medical records were accessed to gather information on their disease and treatments received. Throughout the study, women were consistently reassured of their rights to withdraw at any time without any repercussions.

### Study variables

Data on women’s LLL, menopausal symptoms, general QoL, and endometrial-cancer-specific QoL were collected through various questionnaires administered to all women. These included the Gynecologic Cancer Lymphedema Questionnaire (GCLQ) [23], the Greene Climacteric Scale (GCS) [24], and the European Organization for Research and Treatment of Cancer Quality of Life Questionnaire C30 version 3.0 (EORTC QLQ-C30) [21], alongside the EORTC QLQ–Endometrial Cancer Module 24 (QLQ-EN24) [22]. Specifically, the EORTC QLQ-C30, a cancer-specific questionnaire, was used to measure the general QoL of patients with cancer, while the QLQ-EN24 was developed to enhance the assessment provided by the QLQ-C30 [25]. These specific questionnaires are crucial for evaluating the long-term impact of treatment on QoL, serving as a vital complement to cancer-specific instruments [26]. In addition to these assessments, demographic information and disease specifics were self-reported by the participants, with medical records referenced only after obtaining explicit permission from the women.

#### Lower limb lymphedema

In our study, the assessment of women’s LLL was conducted using the GCLQ [23]. This self-reported instrument comprises 20 items across seven domains, namely heaviness, general swelling, limb-related swelling, infection, aching, numbness, and physical functioning—designed to capture symptoms related to the lower extremities. Each item is rated on a dichotomous scale from 0 (no) to 1 (yes), enabling a total possible score ranging from 0 to 20.

A symptom cluster score was calculated by summing the items, with a threshold set at a total score of 4 or higher as established in a validation study [23]. This cutoff (≥ 4) demonstrated high sensitivity (92.86%) and specificity (83.33%), along with a Cohen’s kappa of 0.759, effectively distinguishing between women with and without LLL [23]. These results further support the GCLQ as a robust, high-quality measure, underscoring the importance of patient-reported surveys in assessing the impact of lymphedema outcomes among EC survivors [27].

The original English version of the GCLQ has demonstrated excellent internal consistency, with a Cronbach’s alpha of 0.95 [23]. The Chinese version of the GCLQ has also been utilized in an intervention study involving Chinese women who underwent gynecological cancer surgery and experienced LLL, examining the effects of diaphragmatic breathing combined with limb training [28].

#### Climacteric symptoms

Climacteric symptoms in women with EC were assessed using the 21-item traditional Chinese version of the GCS for Taiwan [29], which is adapted from the original English version [24]. The GCS recognizes the climacteric period as a complex, multifaceted transition in a woman’s life, marking the shift from reproductive to nonreproductive states and encompassing biological, social, and psychological dimensions [24]. To accurately reflect the distinct etiologies of climacteric symptoms, which span various domains, the GCS evaluates each symptom individually. It comprehensively addresses four primary domains—anxiety, depression, somatic symptoms, and vasomotor symptoms—and includes an additional measure for sexual dysfunction [24]. Each symptom is rated on a scale from 0 (not at all) to 3 (extremely), culminating in a total possible score of 63 across the scale.

The subscales are detailed as follows: the anxiety subscale sums questions 1 to 6 for a possible score of 0–18; the depression subscale covers questions 7 to 11 with a score range of 0–15; the somatic-symptoms subscale includes questions 12 to 18, yielding a score of 0–21; the vasomotor symptoms are calculated from questions 19 and 20 with a total of 0–6; and sexual dysfunction is evaluated by question 21 alone, with a score of 0–3 [24].

The GCS has shown good construct validity, with factor loadings greater than 0.35 [24]. The scale’s internal consistency is robust, particularly among Taiwanese women in perimenopause or postmenopause, demonstrated by a Cronbach’s alpha of 0.82 to 0.89 [30]. Its applicability extends across diverse populations, as evidenced by Australian women with cancer [31], Taiwanese women with breast cancer [32], and Taiwanese women experiencing menopausal syndrome [33], highlighting the GCS’s broad utility in cancer and menopausal studies.

#### General QoL

The comprehensive QoL for women diagnosed with EC was assessed using the 30-item traditional Chinese version of the EORTC QLQ-C30 version 3.0, specifically adapted for Taiwan [34]. This instrument is divided into three dimensions: global health status/QoL, functional status, and symptom status [21]. Within the global health status/QoL dimension, the two items are rated on a scale from 1 (very poor) to 7 (excellent), with higher scores indicating better health status or quality of life.

The instrument further investigates symptom status, which includes a spectrum of symptoms associated with the condition, such as fatigue (3 items), nausea and vomiting (2 items), pain (2 items), dyspnea (1 item), insomnia (1 item), appetite loss (1 item), constipation (1 item), diarrhea (1 item), and financial difficulties (1 item). In contrast, the functional-status dimension addresses various aspects of daily life, covering physical (5 items), role (2 items), emotional (4 items), cognitive (2 items), and social functionality (2 items) [21].

Items on both the functional and symptom scales are scored from 1 (not at all) to 4 (very much). Higher scores on the functional scales indicate better functionality, while higher scores on the symptom scales denote greater symptom severity. The scoring process involves normalizing the raw scores (*RS*), calculated as the average of the items (*RS* = (*I*_*1*_ + *I*_*2*_ + *I*_*3*_ +… + *I*_*n*_) / *n*,). These scores are then transformed: for symptom scales/items and global health status/QoL, the formula used is score = {(*RS*– 1)/*range*} * 100; for functional scales, it is score = {1– (*RS*– 1)/*range*} * 100 [21].

The EORTC QLQ-C30 has demonstrated reliability, with coefficients for its multi-item scales ranging from 0.52 to 0.89 in cancer patients undergoing treatment [21]. Additionally, the moderate size of the interscale correlations validates that the scales assess distinct components of the QoL construct [21]. In a study involving Taiwanese women with breast cancer, the QLQ C-30 version 3.0 showed satisfactory internal consistency for most scales (≥ 0.70), although the scales for physical functioning (0.68) and cognitive functioning (0.53) were exceptions [35]. Furthermore, the QLQ C-30 has been applied in diverse populations, including Polish women with endometrial cancer [22] and Taiwanese women with cervical cancer [36], underscoring its broad applicability.

#### Endometrial cancer–specific QoL

The QoL for women with EC was assessed using the traditional Chinese version of the EORTC QLQ-EN24 [25], adapted from the original English version for use in Taiwan. This instrument is specifically designed to evaluate the QoL impacts associated with EC and its treatments. It comprises two main dimensions: a functional dimension and a symptom dimension [25].

The functional dimension of the EORTC QLQ-EN24 includes three items focusing on sexual interest, activity, and enjoyment, with higher scores indicating better functioning. In contrast, the symptom dimension is broader, encompassing 21 items that address a range of issues: lymphedema (2 items), urological symptoms (4 items), gastrointestinal symptoms (5 items), poor body image (2 items), sexual/vaginal problems (3 items), pain in the back and pelvis (1 item), tingling or numbness (1 item), muscle pain (1 item), hair loss (1 item), and changes in taste (1 item). In this dimension, higher scores signify more severe symptoms [25]. Both dimensions use the scoring methodology of the EORTC QLQ-C30 version 3.0, ensuring consistent measurement.

The reliability of the QLQ-EN24 is supported by acceptable Cronbach’s alpha coefficients for its five multi-item scales, ranging from 0.74 to 0.86 [25]. Test-retest reliability also shows strong correlations, with multi-item scales ranging from 0.81 to 0.92 and single-item scales from 0.72 to 0.97 [25]. In terms of validity, convergent and divergent analyses reveal that most scales of the QLQ-EN24 are weakly correlated with the QLQ-C30 (*r* < 0.40). However, specific items show moderate relationships, such as back/pelvic pain in the QLQ-EN24 with the pain scale of the QLQ-C30 (*r* = 0.57) and the gastrointestinal-symptom scale of the QLQ-EN24 with diarrhea in the QLQ-C30 (*r* = 0.57). The highest correlation observed is between taste change in the QLQ-EN24 and loss of appetite in the QLQ-C30 (*r* = 0.62) [25]. The application of QLQ-EN24 in Taiwan has been well-established [25].

#### Demographic and disease characteristics

The demographic data collected included age, body mass index (BMI), marital status, education level, and menopausal status. Disease-related information encompassed the time since diagnosis, American Joint Committee on Cancer (AJCC) stage, treatment types, and surgical procedures. A dedicated sheet was designed by the research team to document this demographic and disease information.

To assess the participants’ BMI, equipment at the gynecological oncology clinic was utilized to measure their weight and height after obtaining their consent to participate in the study. BMI was then categorized based on the standards provided by the Health Promotion Administration (HPA), Taiwan. The categories were defined as follows: underweight (BMI < 18.5 kg/m^2^), normal (18.5 kg/m^2^ ≤ BMI < 24 kg/m^2^), overweight (24 kg/m^2^ ≤ BMI < 27 kg/m^2^), and obesity (BMI > 27 kg/m^2^) [37].

### Statistical analyses

Demographic data, disease information, and questionnaire scores were analyzed using descriptive statistics, including frequencies, means, and standard deviations (*SD*). Questionnaire scores were calculated provided at least 80% of the items were completed. For any unanswered item, the score was prorated based on the mean score of the responded items within the same questionnaire. Differences in demographic and disease characteristics between the LLL and non-LLL groups were evaluated using Chi-square tests and independent-sample *t*-tests. Additionally, differences in scores for the GCS, EORTC QLQ-C30, and QLQ-EN24 between the two groups were assessed using independent-sample *t*-tests. Cohen’s effect size for the *t*-tests was calculated using the formula: *d* = (mean_1_– mean_2_)/*SD*_*pooled*_. The thresholds for effect size were defined as follows: a small effect size at 0.2, a moderate effect size at 0.5, and a large effect size at 0.8 [38]. All statistical analyses were performed using IBM SPSS Statistics for Windows, version 24.0 (IBM Corporation, Armonk, NY, USA), with the significance level set at *p* ≤ 0.05.

## Results

A total of 189 potential participants with EC were initially recruited for the study. However, 83 women declined to participate for various reasons, including potential diagnoses of other cancers, inconvenience due to obesity, excessive fatigue, recurrence of disease, and disability. Additionally, one woman withdrew during the study, citing its complexity. Consequently, 105 women with EC were recruited and ultimately completed the study. The age of the women ranged from 26.70 to 66.98 years, with a mean of 52.35 years (*SD* = 8.31), and the average BMI was 26.51 (*SD* = 5.64, range = 17.47–43.28). The majority, 94 women (89.5%), had stage 1 disease, and most had received only surgical treatment (80%). Approximately 60% of the women underwent a hysterectomy and BSO with bilateral pelvic lymph node dissection (BPLD) or sentinel lymph node dissection. Surgical menopause was noted in 66 women (62.9%). The prevalence of LLL among the participants was 39% (41 out of 105). There were no significant differences in age, BMI, marital status, education level, menopausal status, time since diagnosis, AJCC stage, treatment category, or surgical procedures between the women with LLL and those without. Detailed demographic and disease characteristics of the women are presented in Table 1.

**Table 1:** Demographic and disease characteristics of women with endometrial cancer (*N* = 105)

Variables	Total sample (*N*=105)	No LLL (GCLQ≤3) (*n*=64; 61%)	LLL (GCLQ≥4) (*n*=41; 39%)	Comparison between groups
	Mean (SD) or *n* (%)	Mean (SD) or *n* (%)	Mean (SD) or *n* (%)	*p* ^a^
Demographic characteristics				
Age	52.35 (8.31)	52.09 (8.86)	52.77 (7.46)	0.68
BMI (kg/m^2^)	26.51 (5.64)	26.11 (5.70)	27.14 (5.56)	0.36
Underweight (< 18.5)	2 (1.9)	2 (3.1)	0	0.31
Normal weight (18.5–24)	38 (36.2)	25 (39.1)	13 (31.7)	
Overweight (24–27)	27 (25.7)	13 (20.3)	14 (34.1)	
Obesity (> 27)	38 (36.2)	24 (37.5)	14 (34.1)	
Marital status				
Married/living with partner	77 (73.3)	49 (76.6)	28 (68.3)	0.19
Divorced/widow	13 (12.4)	9 (14.1)	4 (9.8)	
Single	15 (14.3)	6 (9.4)	9 (22.0)	
Education level				
Below junior high school	20 (19.0)	13 (20.3)	7 (17.1)	0.22
High school	37 (35.2)	26 (40.6)	11 (26.8)	
College or beyond	48 (45.7)	25 (39.1)	23 (56.1)	
Menopausal status				
Premenopause/perimenopause	3 (2.9)	3 (4.7)	0	0.37
Menopause	36 (34.3)	22 (34.4)	14 (34.1)	
Surgical menopause	66 (62.9)	39 (60.9)	27 (65.9)	
Disease characteristics				
Time since diagnosis (months)	37.96 (33.72)	38.63 (33.32)	36.92 (34.72)	0.80
AJCC stage				
1 A/1B	94 (89.5)	60 (93.8)	34 (82.9)	0.13
2 A/2B	5 (4.8)	1 (1.6)	4 (9.8)	
3 A/3B	6 (5.7)	3 (4.7)	3 (7.3)	
Treatment category				
Surgery	84 (80.0)	50 (78.1)	34 (82.9)	0.51
Surgery with chemotherapy	10 (9.5)	7 (10.9)	3 (7.3)	
Surgery with radiotherapy	1 (1.0)	0	1 (2.4)	
Surgery with chemo-radiotherapy	10 (9.5)	7 (10.9)	3 (7.3)	
Hysterectomy				
ATH	46 (43.8)	25 (39.1)	21 (51.2)	0.16
LH	29 (27.6)	22 (34.4)	7 (17.1)	
LAVH	29 (27.6)	17 (26.6)	12 (29.3)	
Hysteroscopic excision of residual endometrial tumor	1 (1.0)	0	1 (2.4)	
Salpingectomy and/or oophorectomy				
No	3 (2.9)	2 (3.1)	1 (2.4)	0.71
BSO	85 (81.0)	51 (79.7)	34 (82.9)	
BS	13 (12.4)	7 (10.9)	6 (14.6)	
RS and LSO	2 (1.9)	2 (3.1)	0	
BS and LO	1 (1.0)	1 (1.6)	0	
Enucleation of right ovarian cyst	1 (1.0)	1 (1.6)	0	
Lymph node resection				
No	39 (37.1)	24 (37.5)	15 (36.6)	0.49
BPLD only	33 (31.4)	18 (28.1)	15 (36.6)	
Pelvic lymph node sampling only	3 (2.9)	3 (4.7)	0	
Bilateral pelvic sentinel lymph node dissection only	14 (13.3)	10 (15.6)	4 (9.8)	
Pelvic sentinel lymph node detection only	5 (4.8)	3 (4.7)	2 (4.9)	
Retroperitoneal lymphadenectomy only	4 (3.8)	3 (4.7)	1 (2.4)	
BPLD and PALD	4 (3.8)	1 (1.6)	3 (7.3)	
Excision of right para-aortic node	1 (1.0)	1 (1.6)	0	
Extirpation of left para-aortic nodes and right lower obturator nodes	1 (1.0)	1 (1.6)	0	
Biopsy of bilateral pelvic lymph node and left para-aortic lymph node	1 (1.0)	0	1 (2.4)	

*SD* standard deviation, *BMI* body mass index, *LLL* lower limb lymphedema, *AJCC* American Joint Committee on Cancer, *GCLQ* Gynecologic Cancer Lymphedema Questionnaire, *BSO* bilateral salpingo-oophorectomy, *BS* bilateral salpingectomy, *RS* right salpingectomy, *LSO* left salpingo-oophorectomy, *LO* left oophorectomy, *BPLD* bilateral pelvic lymph node dissection, *PALD* para-aortic lymph node dissection

^*a*^ Independent-sample *t*-tests for age, BMI, and time since diagnosis; Chi-squared tests for all other variables

Analysis of the GCLQ scores in this study revealed an overall mean of 3.42 points (*SD* = 3.53) among all participants. Notably, there was a significant difference between the LLL and non-LLL groups. Women in the LLL group reported higher mean scores (*mean* = 7.12, *SD* = 2.77) compared to those in the non-LLL group (*mean* = 1.05, *SD* = 1.05), with this difference being statistically significant (*p* < 0.001) and yielding a large effect size (*d* = 3.18). Additionally, the LLL group consistently reported higher scores across all symptoms assessed by the GCLQ, with each symptom also exhibiting large effect sizes. For the validation of the Chinese version of the GCLQ used in this study, six nursing experts reviewed the 20-item scale, confirming its content validity with an excellent scale-level content validity index (S-CVI) of 1.0 using the averaging approach. Furthermore, the scale demonstrated good internal consistency, as evidenced by a Cronbach’s alpha of 0.84. Detailed GCLQ scores for the study population are included in Table 2.

**Table 2:** Scores of GCLQ and its subscales of women with endometrial cancer (*N* = 105)

Symptom clusters	Total sample (*N* = 105)	LLL (*n* = 41)	Non-LLL (*n* = 64)	Comparison between groups	Effect size
	Mean (SD)	Mean (SD)	Mean (SD)	*p* ^a^	d
GCLQ _ total score	3.42 (3.53)	7.12 (2.77)	1.05 (1.05)	0.000	3.18
Physical functioning	0.68 (1.11))	1.51 (1.33)	0.14 (0.39)	0.000	1.55
Numbness	1.01 (1.25)	2.12 (1.25)	0.30 (0.52)	0.000	2.07
General swelling	0.49 (0.75)	1.05 (0.86)	0.13 (0.33)	0.000	1.55
Limb-related swelling	0.13 (0.39)	0.29 (0.56)	0.03 (0.18)	0.001	0.69
Infection	0.30 (0.64)	0.66 (0.82)	0.08 (0.32)	0.000	1.02
Heaviness	0.30 (0.46)	0.68 (0.47)	0.06 (0.24)	0.000	1.78
Aching	0.50 (0.50)	0.80 (0.40)	0.31 (0.47)	0.000	1.10

*SD* standard deviation, *LLL* lower limb lymphedema, *GCLQ* Gynecologic Cancer Lymphedema Questionnaire

^*a*^ Independent-sample *t*-tests for the scores of the GCLQ and its subscales

In this study, the average GCS total score among the sample of women with EC was 11.00 points (*SD* = 7.32). Further analysis showed that women with LLL experienced more severe symptoms, with an average GCS score of 14.10 points (*SD* = 7.80), compared to those without LLL, who had an average score of 9.02 points (*SD* = 6.30). This difference resulted in a large effect size (*d* = 0.73). Significant differences were observed in the domains of anxiety, depression, and somatic and vasomotor symptoms, with the LLL group reporting higher and more pronounced symptoms, exhibiting moderate to large effect sizes. However, no significant differences were found in the sexual dysfunction scores between the two groups. The GCS demonstrated good internal consistency, with a Cronbach’s alpha coefficient of 0.86. The subscale scores for the GCS were as follows: anxiety at 0.80, depression at 0.69, somatic symptoms at 0.75, and vasomotor symptoms at 0.74.

In this study, the overall sample reported a mean score of 70.63 points (*SD* = 20.02) on the global health status subscale of the EORTC QLQ-C30. Specifically, women with LLL had a slightly lower average score of 66.26 points (*SD* = 24.29), compared to 73.44 points (*SD* = 16.33) for those without LLL; however, this difference was not statistically significant. The EORTC QLQ-C30 demonstrated robust internal consistency, with Cronbach’s alpha coefficients of 0.86 for the global health status/QoL, 0.84 for the functional scales, and 0.75 for the symptom scales.

In the EORTC QLQ-C30 functional subscales, role functioning achieved the highest mean score of 97.30 points (*SD* = 10.87) across the overall sample, while cognitive functioning recorded the lowest at 83.33 points (*SD* = 17.45). Notably, significant differences with moderate to large effect sizes were observed across all functional subscales between the two groups. Women with LLL reported poorer outcomes in physical, role, emotional, cognitive, and social functioning compared to those without LLL.

In terms of symptom-related issues, insomnia (*mean* = 23.17, *SD* = 24.51) and fatigue (*mean* = 18.31, *SD* = 18.10) emerged as the most significant symptoms experienced by the overall sample. Additionally, women with LLL reported significantly higher levels of fatigue, pain, dyspnea, and financial difficulties, indicating more severe symptoms compared to those without LLL, and these differences were characterized by moderate to large effect sizes.

Out of the 105 women with EC, 37 (35.23%) resumed sexual activity posttreatment, including 23 from the non-LLL group and 14 from the LLL group. Analysis showed no significant differences in the QLQ scores on the -EN24 functional subscales, which assess sexual interest, sexual activity, and sexual enjoyment, between the two groups.

Excluding the sexual/vaginal problems subscale, the most commonly reported symptoms across the cohort were muscular/joint pain (*mean* = 25.71, *SD* = 26.66), tingling/numbness (*mean* = 20.32, *SD* = 24.68), and back/pelvic pain (*mean* = 19.37, *SD* = 25.23). Women with LLL reported significantly more severe symptoms, including lymphedema, urological and gastrointestinal issues, body image concerns, back/pelvic pain, tingling/numbness, muscular/joint pain, and hair loss, compared to those without LLL, with moderate to large effect sizes. Comparative mean scores for the GCS, EORTC QLQ-C30, and QLQ-EN24 across the LLL and non-LLL groups are detailed in Table 3. The QLQ-EN24 demonstrated good internal consistency in this study, with Cronbach’s alpha coefficients of 0.64 for the functional dimension and 0.77 for the symptom dimension.

**Table 3:** Mean GCS, EORTC QLQ-C30, and QLQ-EN24 scores by LLL and non-LLL groups

Variables	Total sample (*N* = 105)	LLL (*n* = 41)	Non-LLL (*n* = 64)	Comparison between groups	Effect size
	Mean (SD)	Mean (SD)	Mean (SD)	*p* ^a^	d
GCS scores	11.00 (7.32)	14.10 (7.80)	9.02 (6.30)	0.00	0.73
Anxiety	3.16 (2.91)	3.93 (2.82)	2.67 (2.88)	0.03	0.44
Depression	2.30 (1.95)	2.78 (2.17)	1.98 (1.73)	0.04	0.42
Somatic (physical) symptoms	3.37 (2.81)	4.63 (3.40)	2.56 (2.01)	0.00	0.78
Vasomotor symptoms	0.69 (1.18)	1.10 (1.50)	0.42 (0.83)	0.01	0.60
Sexual dysfunction	1.49 (1.28)	1.66 (1.24)	1.38 (1.30)	0.27	0.22
EORTC QLQ-C30 questionnaire scores					
Global health status/QoL					
Global health status	70.63 (20.02)	66.26 (24.29)	73.44 (16.33)	0.10	–0.36
Functional subscales					
Physical functioning	92.70 (9.72)	87.15 (11.68)	96.25 (6.04)	0.00	–1.05
Role functioning	97.30 (10.87)	93.09 (16.66)	100 (0.00)	0.01	–0.41
Emotional functioning	87.94 (13.53)	83.13 (14.73)	91.02 (11.81)	0.01	–0.61
Cognitive functioning	83.33 (17.45)	77.64 (21.61)	86.98 (13.10)	0.02	–0.55
Social functioning	94.13 (12.01)	91.06 (13.99)	96.09 (10.18)	0.05	–0.43
Symptom subscales					
Fatigue	18.31 (18.10)	26.56 (21.36)	13.02 (13.36)	0.00	0.80
Nausea and vomiting	3.33 (9.36)	4.47 (11.80)	2.60 (7.41)	0.32	0.20
Pain	13.17 (16.94)	22.36 (19.94)	7.29 (11.45)	0.00	0.98
Dyspnea	6.03 (13.69)	9.76 (17.07)	3.65 (10.49)	0.04	0.45
Insomnia	23.17 (24.51)	26.02 (27.40)	21.35 (22.51)	0.34	0.19
Appetite loss	4.76 (11.72)	6.50 (13.37)	3.65 (10.49)	0.25	0.24
Constipation	9.84 (18.45)	11.38 (21.87)	8.85 (15.98)	0.50	0.14
Diarrhea	11.43 (19.52)	10.57 (15.70)	11.98 (21.71)	0.72	–0.07
Financial difficulties	4.44 (11.39)	8.94 (14.95)	1.56 (7.10)	0.01	0.68
QLQ-EN24 questionnaire scores					
Functional subscales					
Sexual interest	14.29 (18.98)	14.63 (18.33)	14.06 (19.52)	0.88	0.03
Sexual activity	13.33 (18.83)	10.57 (15.70)	15.10 (20.51)	0.20	–0.24
Sexual enjoyment	38.74 (25.48)^*b*^	42.86 (27.51)^*c*^	36.23 (24.44)^*d*^	0.45	0.26
Symptom subscales					
Lymphoedema	11.27 (17.22)	21.54 (19.81)	4.69 (11.30)	0.00	1.11
Urological symptoms	9.44 (12.40)	13.01 (14.32)	7.16 (10.49)	0.03	0.48
Gastrointestinal symptoms	8.70 (10.22)	11.38 (12.31)	6.98 (8.27)	0.05	0.44
Body image problems	7.62 (20.02)	14.63 (25.87)	3.13 (13.57)	0.01	0.60
Back/pelvic pain	19.37 (25.23)	30.08 (27.69)	12.50 (21.00)	0.00	0.74
Tingling/numbness	20.32 (24.68)	29.27 (27.08)	14.58 (21.31)	0.00	0.62
Muscular/joint pain	25.71 (26.66)	42.28 (26.90)	15.10 (20.51)	0.00	1.17
Hair loss	16.83 (24.95)	24.39 (27.91)	11.98 (21.71)	0.01	0.51
Taste change	3.49 (11.25)	5.69 (14.72)	2.08 (8.13)	0.16	0.32
Sexual/vaginal problems	32.73 (24.70)^*b*^	34.92 (35.10)^*c*^	31.40 (16.29)^*d*^	0.73	0.14

SD standard deviation, *LLL* lower limb lymphedema, *GCS* Greene Climacteric Scale, *EORTC QLQ-C30* European Organization for Research and Treatment of Cancer Quality of Life Questionnaire Core 30, *QLQ-EN24* Quality of Life Questionnaire-Endometrial Cancer Module

^*a*^ Independent-sample *t*-tests, ^*b*^*n* = 37, ^*c*^*n* = 14, ^*d*^*n* = 23

## Discussion

In this study, approximately 39% of women with endometrial cancer (EC) were identified as having LLL when assessed using the GCLQ. This finding mirrors the prevalence reported in a previous study [7], which also found that 39% of women experienced LLL (11%) or lower limb swelling (28%) following EC treatment. The consistency between these findings underscores the significant incidence of LLL among women treated for EC, highlighting the importance of recognizing and managing this common post-treatment complication.

In this study, the mean GCLQ score for the total sample was 3.42, slightly lower than the 4.4 reported in a previous North Carolina study of 50 women with EC, including 27 White and 23 Black women [27]. This discrepancy may stem from differences in sample size and demographics. Aligning with prior research on Korean women with primary EC, which showed significant differences in total GCLQ scores between groups with and without lower extremity edema (LEE) (*p* < 0.001) [39], our findings also indicated significant differences in GCLQ scores between women with LLL (*mean* = 7.12) and those without (*mean* = 1.05), reflecting a large effect size. Similarly, another study involving 58 women with gynecological cancer found that those with lower extremity lymphedema (LLE) had significantly higher GCLQ scores (*mean* = 8.89) compared to those without LLE (*mean* = 1.63) [23]. Our results also showed that all symptom cluster scores were significantly higher in the LLL group, supporting earlier findings [23]. These results underscore the GCLQ’s effectiveness in distinguishing between women with and without LLL.

Women diagnosed with EC often experience climacteric symptoms following a hysterectomy and bilateral salpingo-oophorectomy, especially if they were premenopausal prior to surgery. In our study, over 60% of the participants underwent surgical menopause due to their cancer treatment. Our results indicate that women with EC who also suffered from LLL reported significantly more severe climacteric symptoms, including anxiety, depression, and somatic and vasomotor symptoms, compared to those without LLL. Direct comparisons are challenging, as few studies have specifically explored the impact of LLL on menopausal symptoms in women with EC. However, a previous two-year longitudinal study involving 132 women with EC found that menopausal symptoms, the most disabling sequelae, peaked one year after surgery and persisted at the two-year mark [13]. Future research focusing on women with EC who have experienced LLL should adopt a longitudinal design to specifically track the progression of climacteric symptoms and compare these findings with those who did not suffer from LLL.

In our study, the mean score on the global health status subscale of the EORTC QLQ-C30 for the total sample was 70.63 points, closely aligning with the findings of Karataşlı et al. [40], who reported a mean score of 71 points among nonobese women with EC and a BMI less than 30 kg/m^2^. The non-LLL subgroup in our study scored slightly higher, with a mean of 73.44, compared to 66.26 for the LLL subgroup, though this difference was not statistically significant. Few studies focus specifically on the impact of LLL on the global health status of women with EC, making direct comparisons with other studies challenging. However, a study by Wedin et al. [41] involving 235 women with early EC, using the SF-36, EQ-5D-3 L, and LYMQOL (Lymphoedema quality of life questionnaire), found no significant differences in generic health-related QoL or LYMQOL global scores between women with and without LLL. It did, however, reveal significant impairments in function, appearance/body image, and physical symptoms in women with LLL, but not in the emotion/mood domain [41]. This suggests that while the global health status differences between women with and without LLL post-EC treatment are not statistically significant, there is a noticeable trend toward lower status in those with LLL. Future research should involve larger sample sizes and use sensitive QoL measures to detect specific changes associated with LLL. Additionally, employing mixed-methods designs is crucial to comprehensively understand the experiences and challenges faced by women post-EC treatment.

In our study, role functioning emerged as the highest-scoring domain on the EORTC QLQ-C30 functional subscales, aligning with findings from studies on nonobese women with EC [40] and those undergoing vaginal brachytherapy (VBT) [42], both of which also reported the highest scores in this domain. Conversely, cognitive functioning scored the lowest in our study, diverging from prior research that included 201 Turkish women with EC who had completed primary surgery and their last treatment at least 12 months earlier [40], and 55 German women with EC, with or without VBT [42], where emotional functioning typically scored the lowest. However, the mean score for cognitive functioning in our study (*mean* = 83.33) was slightly higher than that reported for 60 nonobese Turkish women with EC (*mean* = 80) [40] and 29 German women undergoing VBT (*mean* = 82.8) [42]. These discrepancies may be attributed to differences in sample size and treatment modalities.

Women in the LLL subgroup experienced significantly poorer outcomes across all functional subscales of the EORTC QLQ-C30, including physical, role, emotional, cognitive, and social functioning, compared to those in the non-LLL subgroup. This reflects the broader implications of LLL on daily life and social interactions. Ryan et al. [17] conducted structured interviews with 82 women with gynecological cancer who developed LLL and found that LLL affected their lifestyles, including financial burdens related to the cost of compression garments and complex physical therapy. Additionally, LLL necessitated clothing alterations to accommodate changes in self-image, comfort, and appearance. LLL also led to modifications in daily activities, such as difficulties with standing or sitting for extended periods. Moreover, the condition resulted in restricted social activities and required various precautions [17]. Beyond that, our study found that emotional well-being was adversely affected, with higher levels of anxiety and depression observed among women with LLL compared to those without. This aligns with findings from Kusters et al. [43], which highlighted the negative impact of LLL on anxiety and depression symptoms following gynecological cancer treatment.

In our study, insomnia and fatigue emerged as the most severe symptoms reported by all women with EC, as indicated by the EORTC QLQ-C30 symptom subscales. This observation aligns with previous research on nonobese women with EC [40] and women undergoing VBT [42]. Additionally, a survey study involving 1,029 women with gynecological cancers identified fatigue (60.6%) and sleep disturbances (54.9%) as the most common health issues encountered before, during, and after treatment [44]. Although fatigue and insomnia are often considered normal reactions to cancer diagnosis and treatment [45, 46], distinguishing these as independent symptoms rather than as manifestations of other conditions such as depression or anxiety remains challenging. Furthermore, a cross-sectional study of 134 Taiwanese women with EC found that sleep quality was the most significant predictor of fatigue [47], emphasizing the intricate relationship between sleep and overall well-being in women with EC.

In our cohort, women with LLL reported significantly increased symptoms of fatigue, pain, dyspnea, and financial difficulties compared to those without LLL. Fatigue was notably the most severe symptom for those with LLL, contributing significantly to reduced QoL and accounting for 11% of the variance in QoL after gynecological cancer surgery [48]. Advanced LLL can lead to pain, reduced muscle strength, and increased susceptibility to infections [49] and can have a considerable impact on daily life. Challenges include limited ability to stand or sit for extended periods and reduced participation in social and daily activities due to concerns about appearance and physical discomfort [17]. Lymphedema-related fluid retention can further lead to weight gain and decreased physical strength, exacerbating symptoms like dyspnea. The financial implications are also significant, with about 22% of women incurring extra costs related to managing LLL, including expenses for compression garments and complex physical therapy [17]. These findings underscore the need for targeted interventions to manage symptoms and improve the quality of life in this patient population.

In this study, approximately 35% of women with EC resumed sexual activity posttreatment across our entire study population. This rate is lower compared to a Turkish study, where about 69.1% of EC survivors engaged in sexual intercourse three months after the last treatment and 26.4% remained sexually inactive [40], suggesting potential cultural influences or the impact of a smaller sample size. Additionally, the Turkish study reported a high prevalence of sexual dysfunction, approximately 95%, underscoring the need for increased attention to sexual health issues in EC survivors [40].

Further analysis in our study using the QLQ-EN24 functional subscales showed no significant differences in sexual interest, activity, or enjoyment between the LLL and non-LLL groups. Additionally, our findings indicated no significant variations in sexual dysfunction, as measured by the GCS, or in sexual/vaginal problems, as assessed by the QLQ-EN24 symptom subscale, between the groups. Dunberger et al. [50] corroborated these results, finding no significant differences in sexual satisfaction between gynecological cancer survivors with and without LLL. Despite the lack of significant differences, sexual/vaginal problems were prevalent among both the total sample and individual groups in our study, indicating these issues are widespread among women with EC. Compared to the general population, a greater proportion of EC survivors face sexual dysfunctions, including problems with sexual desire, arousal, painful intercourse, and reduced orgasm intensity [51]. These findings emphasize the prevalence of sexual issues among EC survivors and the necessity for more comprehensive management strategies, regardless of lymphedema status.

When assessing cancer-specific QoL for women with EC using the QLQ-EN24, our study found that muscular/joint pain was the most frequently reported symptom across the entire sample, as indicated by the symptom subscales of the QLQ-EN24. This finding aligns with previous research on EC survivors that also used the EORTC QLQ-EN24 [25, 52]. Additionally, our study revealed that women with LLL reported significantly more severe symptoms, including lymphedema, urological and gastrointestinal issues, body image concerns, back/pelvic pain, tingling/numbness, hair loss, and the aforementioned muscular/joint pain, compared to those without LLL. These findings are consistent with those of Dunberger et al. [50], who used a mixed-method study design for gynecological cancer survivors and observed significantly lower QoL and greater daily life activity impairments in survivors with LLL compared to those without, particularly affecting mobility, finances, appearance, and self-image.

This study’s limitations include a small sample size composed of women recruited by convenience from a single medical center in Northern Taiwan, which may limit the generalizability of the findings. The cross-sectional design restricts the ability to explore temporal changes in menopausal symptoms, LLL, and QoL. Additionally, the reliance on quantitative data collection methods without incorporating qualitative approaches limits our understanding of the personal experiences and perspectives of women living with LLL.

## Conclusion

This study conclusively shows that LLL significantly impacts the QoL and climacteric symptom severity in women with EC. Women with LLL report exacerbated symptoms in areas such as fatigue, pain, dyspnea, and psychological distress compared to those without LLL. Despite similar rates of resuming sexual activity, LLL markedly impairs physical, role, emotional, cognitive, and social functioning. Looking ahead, it is crucial to incorporate targeted interventions that address the comprehensive impacts of LLL into treatment plans for women with EC. Future research should emphasize long-term studies to further explore the enduring effects of LLL on QoL and develop holistic care strategies that alleviate its impact and enhance overall QoL in this population.

## Data Availability

The data, for privacy and ethical reasons, cannot be shared publicly, but interested parties can request access to the data from the corresponding author in a reasonable manner.

## Abbreviations

EC: Endometrial Cancer
QoL: Quality of Life
SD: Standard Deviation
BMI: Body Mass Index
LLL: Lower Limb Lymphedema
AJCC: American Joint Committee on Cancer
GCLQ: Gynecologic Cancer Lymphedema Questionnaire
GCS: Greene Climacteric Scale
EORTC QLQ-C30: European Organization for Research and Treatment of Cancer Quality of Life Questionnaire Core 30
QLQ-EN24: Quality of Life Questionnaire-Endometrial Cancer Module
BSO: Bilateral Salpingo-Oophorectomy
BS: Bilateral Salpingectomy
RS: Right Salpingectomy
LSO: Left Salpingo-Oophorectomy
LO: Left Oophorectomy
BPLD: Bilateral Pelvic Lymph Node Dissection
PALD: Para-Aortic Lymph Node Dissection
